# Comparative analysis uncovers the limitations of current molecular detection methods for *Fusarium oxysporum* f. sp. *cubense* race 4 strains

**DOI:** 10.1371/journal.pone.0222727

**Published:** 2019-09-23

**Authors:** Freddy Magdama, Lorena Monserrate-Maggi, Lizette Serrano, Daynet Sosa, David M. Geiser, María del Mar Jiménez-Gasco

**Affiliations:** 1 Department of Plant Pathology and Environmental Microbiology, The Pennsylvania State University, University Park, Pennsylvania, United States of America; 2 Escuela Superior Politécnica del Litoral, ESPOL, Centro de Investigaciones Biotecnológicas del Ecuador, Campus Gustavo Galindo, Guayaquil, Ecuador; 3 Escuela Superior Politécnica del Litoral, ESPOL, Facultad de Ciencias de la Vida, Campus Gustavo Galindo, Guayaquil, Ecuador; Franklin & Marshall College, UNITED STATES

## Abstract

*Fusarium oxysporum* f. sp. *cubense* Tropical Race 4 (*Foc* TR4) is threatening banana production worldwide. Despite quarantine efforts, the pathogen continues to spread; thus, early diagnosis plays an essential role for the proper execution of contingency plans. Here, we assess the accuracy of four PCR-based molecular methods described in the literature for the identification and detection of race 4 strains, including Subtropical (*Foc* STR4) and Tropical Race 4 causing Fusarium wilt of banana. We screened a total of 302 isolates using these four markers, and performed phylogenetic analyses, Vegetative Compatibility Group (VCG) testing, sequence comparison, and pathogenicity tests for selected isolates. Our results show that three out of the four markers tested are not reliable for identification of *Foc* STR4 and TR4, as DNA from isolates from Ecuador, pathogenic and nonpathogenic to banana, obtained from different banana cultivars, displayed cross-reaction with these methods; that is, false positives can occur during the diagnostic process for race 4. Phylogenetic analyses, VCG testing, sequence comparison, and pathogenicity tests suggest the presence of non-target *F*. *oxysporum* isolates that share genomic regions with pathogenic strains but lack true pathogenicity to banana. The findings of this work are of foremost importance for international regulatory agencies performing surveillance tests in pathogen-free areas using the current diagnostic methods. We suggest the use of a genetic locus possibly related to virulence, previously identified by T-DNA, and amplified with primers W2987F/ W2987R, for diagnosis of *Foc* TR4 as the most reliable alternative. We urge the adoption of a more holistic view in the study of *F*. *oxysporum* as a plant pathogen that considers the biology and diversity of the species for the development of better diagnostic tools.

## Introduction

Bananas rank amongst the world’s most valuable primary agricultural commodity and are one of the top ten most important staple foods for countries in Southeast Asia, Africa, and Central and South America [1]. In 2013, the combined global production of bananas, including dessert and cooking bananas was approximately 140 million tons with a gross production value of USD $44.75 billion [1]. Most of this production, around 85%, is destined for local trade and family consumption, while the remaining 15% (worth USD $10.13 billion) is exported for the international market [1]. Over 95% of banana worldwide export production relies on a group of cultivars named Cavendish that includes commercially grown varieties such as ‘Grand Naine’ and ‘Williams’ [2]. These cultivars gradually replaced ‘Gros Michel’, the previous banana used for export purposes that succumbed to disease epidemics in Tropical America between 1900 and 1960 by *Fusarium oxysporum* f. sp. *cubense* (*Foc*) race 1 [3], the causal agent of Fusarium wilt of banana or Panama disease [4].

There are three well-known races of *Foc* identified by their pathogenicity to specific clonal triploid banana hosts [5–7]. Race 1 causes disease to 'Gros Michel' (AAA) and can also cause disease on other cultivars like ‘Silk’ (AAB), ‘Pome’ (AAB), ‘Pisang Awak’ (ABB) and ‘Maqueno’ (AAB) [8]. Race 2 affects cooking bananas, especially those in the Bluggoe subgroup (ABB) [9,10]. Race 4 has been known to cause disease on cultivars susceptible to race 1 and 2, and more importantly, members of the subgroup Cavendish (AAA). Race 4 is divided into two types; Subtropical Race 4 (*Foc* STR4), which is restricted to the subtropics were plants are exposed to stress conditions such us low temperatures and drought [7, 11] and Tropical Race 4 (*Foc* TR4), which causes disease under both tropical and subtropical conditions without predisposing factors [12–14]. Tropical Race 4 has the potential to cause catastrophic losses in the monoculture-based banana industry and become a life-changing event for 65 millions of people, particularly smallholder farmers [15].

The race structure in *Foc* is confusing and often inaccurate in delineating strains of *Foc* [16]. Several DNA-based studies have shown that races of *Foc* are not genetic related to each other or share a common ancestor [17–19]. It is currently widely accepted that *Foc* is composed of several independent lineages with a population structure mainly clonal [12, 17–20]. *Foc* diversity has also been assessed by means of vegetative compatibility, which delimits groups (Vegetative Compatibility Groups—VCGs) that align with clonal lineages. *Foc* comprises 24 VCGs [20, 21], distributed differentially across the globe [19]. Among those, the VCG-01213/16 comprises the group of strains referred to as Tropical Race 4 (*Foc* TR4). This race is spreading to new countries in the South part of Asia, including Laos, Vietnam, Myanmar, India and Pakistan, and outside of its native range in South East Asia, affecting also countries in Southern Africa (Mozambique) and Middle East (Oman, Jordan, Lebanon, and Israel) [22, 23]. Recent evidence shows that epidemics caused by *Foc* TR4 are the result of the movement of a single clone [20], although recent research tracking the spread of *Foc* TR4 in the Mekong Delta shows sequence variation within VCG 01213/16 [24]. Symptoms of Fusarium wilt in Cavendish have also been reported to be associated with VCGs 0124 and 0121 [19, 25, 26]. VCG-0121 is genetically closely related to VCG-01213/16 and may cause disease on Cavendish under tropical conditions [27]. In terms of management, there are still no available solutions for the present monoculture-based, large-scale agricultural system in which bananas are produced. Exclusion and prevention remain the most important and only effective measures to prevent the disease in countries where *Foc* TR4 has not been reported [26].

Fusarium wilt diagnosis has traditionally relied on the recognition of symptoms, mainly characterized by wilting and yellowing of the leaves, accompanied by the reddish-brown discoloration of the pseudostem of diseased bananas [28]. However, decisions made on these criteria may hamper all efforts for disease control since the pathogen has a long incubation period. That is, by the time symptoms are observed in the field, which may take several weeks, the pathogen can be dispersed to new areas. *Foc* can also remain undetected infecting asymptomatic hosts as an endophyte, or colonizing soil as a saprotroph [29]. Therefore, early detection based on DNA tools constitutes a key component for the efficient execution of contingency plans, including quarantine and eradication procedures. Over the last decade, several molecular methods have been developed for detection of race 4, including *Foc* STR4 and TR4 strains [30–38]. Among all, one PCR-based method, using the specific primers *Foc*TR4-F/*Foc*TR4-R has been adopted by the International Regional Organization for Agricultural Health (OIRSA) and the Food and Agriculture Organization of the United Nations (FAO) in their contingency plans for Latin America and Caribbean countries [31, 39–41]. This particular methodology targets *Foc* TR4 strains only (VCG-01213), due to the major risk they represent to Cavendish bananas. This molecular tool goes beyond pathogen detection on symptomatic tissue, and it is also implemented for detecting *Foc* TR4 in asymptomatic plants, soil, and water [20, 42]. However, all the proposed methods have been developed using only pathogenic isolates with few, if any, nonpathogenic isolate included in validation assays. Although the *Fusarium oxysporum* species complex (FOSC) entails pathogenic and nonpathogenic strains [29, 43], studies suggest that greater diversity is found in endophytic and saprotrophic populations, colonizing plants or present in the soil, respectively [44–46]. Considering that the diversity of *F*. *oxysporum* populations has been overlooked and there is a large number of methodologies available for *Foc* race 4 diagnostics, it is imperative to determine which one of these tests provide the most accurate and reliable results for activities related to monitoring and quarantine of *F*. *oxysporum* populations pathogenic to banana.

The main goal of this research was to assess the specificity of four detection methods previously developed for race 4 diagnostics, including STR4 and TR4 [30–33]. In this study, we screened 302 isolates obtained from banana, including pathogenic and endophytic, collected from Ecuador following a PCR approach. The genetic relationship of representative strains used in this study was analyzed by phylogenetic analysis. Validation of the PCR-based screening results included VCG analysis, sequence comparison, pathogenicity testing and detection of selected endophytes from artificially infested soil and infected banana plants of two cultivars.

## Materials and methods

### Fungal collection and DNA extraction

**Fig 1:**
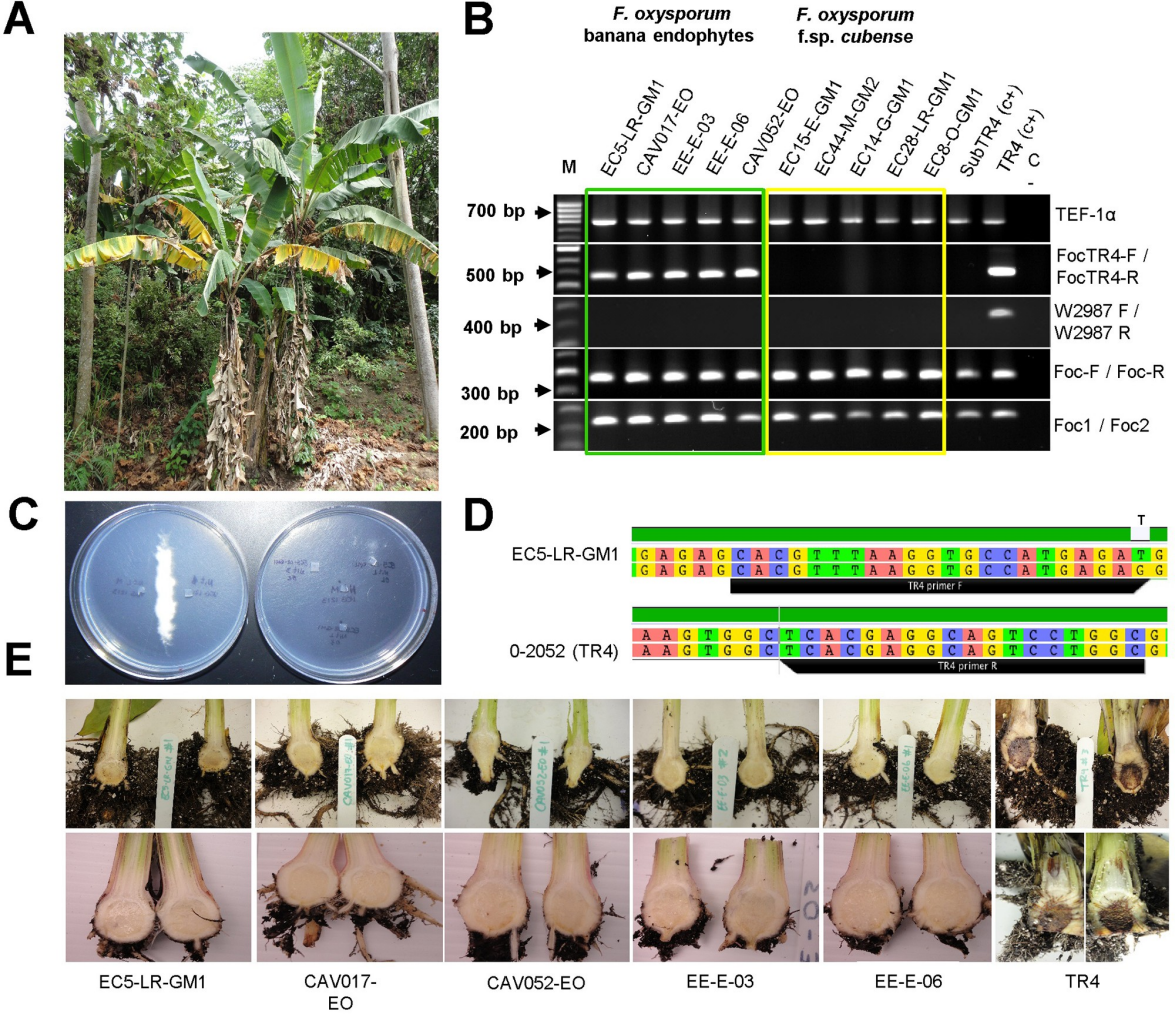
Validation of molecular detection methods for *F*. *oxysporum* f. sp. *cubense* Race 4. (A) ‘Gros Michel’ banana plants showing symptoms of Fusarium wilt. (B) False positives for *F*. *oxysporum* f. sp. *cubense* (*Foc*) -Subtropical and Tropical Race 4- revealed by gel electrophoresis of PCR products generated using DNA from endophytic (nonpathogenic) and pathogenic (race 1) *F*. *oxysporum* isolates. C) Vegetative compatibility group (VCG) test between the isolate EC5-LR-GM1 and the tester strain belonging to VCG-01213 (*Foc* TR4) showing no heterokaryon formation relative to the positive control (left figure). (D) Sequence comparison of segment in the IGS region used for the development of the primer set *Foc*TR4-F/*Foc*TR4-R. All five endophytes associated with false positives reactions shared the same features. (E) Pathogenicity test results showing no symptoms of necrosis in the rhizome of ‘Gros Michel’ and Cavendish 'Gran Naine' banana plantlets 30 days post inoculation (dpi) with the five *F*. *oxysporum* endophytes.

A collection of 302 isolates was analyzed in this study. The collection included 292 *F*. *oxysporum* isolates, including 130 putative pathogenic isolates obtained from symptomatic ‘Gros Michel’ banana plants showing Fusarium wilt symptoms (Fig 1A), and 162 putative endophytic isolates, obtained from asymptomatic Cavendish banana cultivars and other local banana cultivars from several provinces in the coastal area of Ecuador including Esmeraldas, Manabí, Guayas, Los Ríos, El Oro, Santo Domingo and Bolivar (S1 Table). Sampling sites included backyards, home-gardens, roadsides, abandoned farms and banana plantations. Sampling on private land was conducted after granted permission by the owner. Ten isolates of three other *Fusarium* species, also obtained from the roots of banana plants, were included in this study for comparison purposes. In addition, the reference strains O-2052 (VCG-01213), O-1966 (VCG-0120), and O-1968 (VCG-0123), obtained from the Fusarium Research Center at The Pennsylvania State University, and characterized as *Foc* TR4, STR4 and race 1, respectively, were included in this study. This study did not involve endangered species.

Single-spore isolates were grown in potato dextrose broth medium (PDB, Sigma-Aldrich^®^, Saint Louis, USA) for approximately 7 days at 28°C in an orbital environ shaker-incubator (Lab-line instruments Inc., IL, USA) at 120 rpm. Fresh mycelium was harvested and dried out using autoclaved filter paper (Whatman^™^, Amsterdam, UK) of which 200 mg were used for genomic DNA extraction using the DNeasy^®^ plant mini kit (Qiagen, Maryland, USA) following the manufacturer’s guidelines. The concentration and quality of the extracted DNA samples were estimated using a Nanodrop spectrophotometer-2000^™^ (Thermo Fisher Scientific, Wilmington, USA) followed by gel electrophoresis. The amplification of the translation elongation factor one-alpha (TEF) gene was performed to assess the quality of the DNA extracted for all isolates and to corroborate their identity as *F*. *oxysporum* by comparing their TEF sequences to the Fusarium-ID database [47], and used for the phylogenetic analysis (specified in the section below).

### Validation of molecular detection methods

A PCR-based screening was conducted using DNA from all isolates of our collection and four sets of primers; two targeting *Foc* STR4 strains, and two exclusively targeting *Foc* TR4. All the sequences of the primer sets used in this study are described in Table 1. PCR reactions were carried out using Choice Taq Mastermix^™^ (Denville Scientific Inc., Massachusetts, USA) on 25 μL containing 1X reaction buffer, 0.5 μM of each primer, 5 ng of DNA, and sterile deionized water. The cycling conditions used for the primer sets *Foc*1/*Foc*2, *Foc*-F/*Foc*-R, *Foc*TR4-F/*Foc*TR4-R and W2987-F/W2987-R, were the same previously described by Lin et al. [30], Li et al. [32], Dita et al. [31], and Li et al. [33]. DNA from the strains O-2052 and O-1966 was used as template for positive controls for *Foc* TR4 (VCG-01213) and *Foc* STR4 (VCG-0120), respectively. Sterile deionized water was used as negative control.

**Table 1:** Molecular markers and primers used in this study.

Target taxa	Primer	Target region ^a^	*F*. *oxysporum* f.sp. *cubense* race	Sequence (5'-3')	Band size (bp)	Reference
*F*. *oxysporum* f. sp. *cubense*	*Foc*1	OPA02_404_ (RAPD)	ST4 and TR4	CAGGGGATGTATGAGGAGGCT	242	[30]
	*Foc*2			GTGACAGCGTCGTCTAGTTCC		
*F*. *oxysporum* f. sp. *cubense*	*Foc*-F	S22 (SCAR)	ST4 and TR4	ATATGAATGACTCGTGGCACG	364	[32]
	*Foc*-R			GCTGGG AATGCGACGGTAT		
*F*. *oxysporum* f. sp. *cubense*	*Foc*TR4-F	IGS	TR4	CACGTTTAAGGTGCCATGAGAG	463	[31]
	*Foc*TR4-R			GCCAGGACTGCCTCGTGA		
*F*. *oxysporum* f. sp. *cubense*	W2987-F	Gene coding for hypothetical protein	TR4	TGCCGAGAACCACTGACAA	452	[33]
	W2987-R			GCCGATGTCTTCGTCAGGTA		
*Fusarium* species	ef1	TEF	_	ATGGGTAAGGARGACAAGAC	700	[47]
	ef2			GGARGTACCAGTSATCATGTT		
*F*. *oxysporum*	CNL12	IGS	_	CTGAACGCCTCTAAGTCAG	2,600	[48]
	CNS1			GAGACAAGCATATGACTACTG		
	PN22	IGS	_	CAAGCATATGACTACTGGC	_	[49]
	CNSa	IGS	_	TCTCATRTACCCTCCGAGACC	_	[50]
	INL11	IGS	_	AGGCTTCGGCTTAGCGTCTTAG	_	[50]
	U:49–67	IGS	_	AATACAAGCACGCCGACAC	_	[48]
	PNFo	IGS	_	CCCGCCTGGCTGCGTCCGACTC	_	[51]

^a^ RAPD = Radom amplified polymorphic DNA; SCAR = Sequence characterized amplified region; IGS = Intergenic Spacer Region of the rDNA; TEF = Translation elongation factor 1-α

### Phylogenetic analyses

Phylogenetic analyses based on the TEF and the intergenic spacer region of the ribosomal DNA (IGS) were carried out to elucidate the phylogenetic relationship of selected 66 endophytic isolates representative of the sampled sites including five *F*. *oxysporum* endophytes EC5-LR-GM1, CAV017-EO, EE-E-03, EE-E-06, CAV052-EO that generated false positives with the specific primers *Foc*TR4-F/*Foc*TR4-R. The TEF region was amplified using the primer set ef1/ef2 following the PCR conditions recommended by Geiser et al. [47], and the IGS region using the primer set CNL12/CNS1 following the PCR conditions recommended by Appel and Gordon [48]. PCR reactions were carried out in 25 μL using 1X reaction buffer of Choice Taq Mastermix^™^ (Denville Scientific Inc., Massachusetts, USA), 0.5 μM of each primer, 5 ng of gDNA and sterile deionized water. The resulting PCR products were visualized by gel electrophoresis and purified using ExoSAP-IT^™^ (USB Affymetrix Corporation, Cleveland, USA) following the manufacturer’s instructions. All PCR products were sequenced by the Genomic Core facility of The Pennsylvania State University, University Park, PA, USA. TEF regions were sequenced using the same primers as for the PCR amplification while complete sequences for the IGS region were generated using primers PN22 [49], CNSa, iNL11 [50], U: 49–67 [48] and PNFo [51]. For both data sets, the alignments and editing of sequences were performed using the software GENEIOUS R8 v 8.0.5 (Biomatters, Auckland, NZ).

Phylogenies based on Maximum Parsimony (MP), Maximum Likelihood (ML) and Bayesian (BS) methods were inferred for the different data sets using PAUP v 4.0a [52], RAxMLGUI v 1.31 [53], and MrBayes v 3.2.6 [54], respectively. The heuristic search option with 1,000 random addition sequences and tree bisection-reconnection branch swapping was used to infer the most-parsimonious tree. Gaps were treated as missing data. The Consistency Index (CI) and Retention Index (RI) were calculated to indicate the amount of homoplasy present. Maximum likelihood reconstruction was run using the thorough bootstrap analysis and the ML search with 1,000 replicates. The Generalized Time Reversible Model with a Gamma distribution (GTRGAMMA) was used as the model for nucleotide substitution. Bayesian inference was run with a 10,000,000 generation Monte Carlo Markov chain method with a burn-in of 100,000 trees. The General Time Reversible model was selected as the most appropriate nucleotide evolution model for Bayesian analysis. Convergence and the effective sample size (ESS) were checked for each run. The TEF tree was rooted with *Fusarium foetens* NRRL 31847 and the IGS tree with *F*. *foetens* NRRL 31852. For comparative purposes, TEF and IGS sequences of all clonal lineages of *Foc* described by Fourie et al. [19] were incorporated into the analyses. In addition, putative race 1 *Foc* isolates EC15-E-GM1, EC44-M-GM2, EC14-G-GM1, EC28-LR-GM1 and EC8-O-GM1, obtained from symptomatic ‘Gros Michel’ bananas from Ecuador, were also included in this study. Sequences generated in this research were deposited in the GenBank database under accession numbers MK754073- MK754148 (TEF) and MK783034- MK783108 (IGS).

### Determination of VCG group

The five isolates that generated a positive PCR amplification for *Foc* TR4 with the specific primers *Foc*TR4-F/*Foc*TR4-R were also tested for their capability to form a stable heterokaryon with *nit* mutants of the tester strains O-2052, O-1966, and O-1968, associated to the VCGs 01213, 0120 and 0123, respectively. Nitrate non-utilizing (*nit*) mutants were generated on media supplemented with potassium chlorate (KClO3, Sigma-Aldrich^®^, Saint Louis, USA) at 4.5% [19], and incubated for 8–15 days at 27 °C. Colony mutants with a weak growth and no aerial mycelium were further characterized as *nit*-1, *nit*-3 or *nit*-M on media containing one of three sources of nitrogen [55–56]. *Nit*-mutants of each isolate were checked for self-incompatibility. VCG complementation was considered positive when the pairing of two *nit*-mutants resulted in dense aerial growth at the contact zone in minimal medium [55].

### Pathogenicity testing

The five isolates used for VCG determination, were also employed to prepare spore suspensions for inoculation of banana plants. The reference strains O-2052 (VCG-01213), O-1968 (VCG-0123), characterized as *Foc* TR4 and R1, were used as positive and negative controls, respectively. For this, five plugs taken from the margin of actively growing cultures were inoculated in two 1,000-ml graduated laboratory bottles (VWR^®^, Radnor, USA) containing 500 ml of autoclaved PDB. Two extra bottles were prepared containing only the liquid media for negative controls. Inoculated PDB was incubated at room temperature during 12 days in a shaker-incubator (Lab-line instruments Inc., IL, USA) at 120 rpm. Spore suspensions were passed through filter paper (Whatman^™^, Amsterdam, UK) and adjusted to 1x10^6^ conidia/ml before inoculation. Tissue-cultured banana plants approximately six weeks old and 25-cm high, from two cultivars (‘Gros Michel’ and Cavendish ‘Gran Naine’), were inoculated with spore suspensions of each isolate following a root-dipping method [31]. Inoculated plants were placed in pots containing a mixture of autoclaved potting soil and potting mix in a 1:1(w/w) ratio. The experiments were performed following a completely randomized design with four replicates per treatment for the Cavendish experiment and three replicates per treatment for the ‘Gros Michel’ experiment, including positive and negative controls. Both experiments were conducted twice with independently produced inoculum and banana plants. Plants were incubated in growth chambers (Conviron^®^, Winnipeg, Canada) with environmental conditions set at 28°C, 80% relative humidity, and 16/8 h light/dark photoperiod. After 30 days, plants were removed from their pots and cut in half using a sterilized scalpel for internal disease assessment. Internal discoloration of the rhizome was recorded using a five-point rating scale according to Ssali et al. [57] with some modifications where 0 = no discoloration, 1 = 1–15% tissue showing discoloration, 2 = 16–33% tissue showing discoloration, 3 = 34–50% tissue showing discoloration, 4 = more than 50% tissue showing discoloration, and 5 = totally decayed. The disease index (DI) was also calculated based on the formula DI = [Ʃ(score in the scale x frequency)/(total number of plants x maximum class in the scale)] x 100. Isolation of the inoculated strains was performed at the end of the experiments, placing surface disinfested plant tissues, from the rhizome and pseudostem of plants, in petri dishes containing Nash semi-selective media [58].

### Assays for *in-planta* and soil detection

Banana tissue and inoculated soil derived from the pathogenicity tests, were subjected to *Foc* race 4 detection using the same sets of primers evaluated above. Upon completion of the pathogenicity tests, 30 days after inoculation, samples from each banana plant, from both cultivars, were taken from the rhizome and pseudostem using a sterilized scalpel. Samples from the pseudostem were taken 5 cm above the base of each plant. From each sample, three-subsamples of fresh tissue, 20 mg each, were used to extract total DNA using the DNeasy^®^ plant mini kit (Qiagen, Maryland, USA) following the manufacturer’s instructions. For soil analyses, a composite random sample of 1g of soil per treatment, from both experiments, was collected for DNA extraction and subjected to analysis. Total DNA was extracted using the PowerSoil^®^ DNA isolation kit (MO BIO Laboratories, Inc., Carlsbad, CA), using 0.25 gr of soil, following the manufacturer's guidelines. PCR cycling conditions were the same as the ones used with pure mycelia. In order to increase sensitivity, a nested-PCR was included into our analysis for processing plant materials, considering the IGS region as the primary target following the use of the primer set *Foc*TR4-F/*Foc*TR4-R for specific detection of *Foc* TR4. For soil detection, the primer set *Foc*TR4-F/*Foc*TR4-R was used in a duplex PCR with the primer set ef1/ef2. Multiplex PCR was used to detect fungal DNA in one single reaction and discriminate false negatives in soil samples. In addition to positive and negative controls, a sample containing DNA obtained from pure fungal mycelium (5 μL with approximately 20 ng/μL) from the O-2052 (TR4) strain, diluted in a background of total soil gDNA, was also incorporated into our analysis for comparison purposes.

## Results

### PCR-based methods for *Foc* race 4 identification

Among the four sets of primers evaluated, positive PCR reactions were obtained with three of them, including *Foc*-1/*Foc*-2 [30], *Foc*-F/*Foc*-R [32], and *Foc*TR4-F/*Foc*TR4-R [31] when tested with the target DNA (Fig 1B). For the primer sets *Foc*-1/*Foc*-2 and *Foc*-F/*Foc*-R, which target both *Foc* STR4 and TR4, 187 isolates collected from the coastal area of Ecuador resulted in positive amplifications from the 302 fungal isolates, similar to the positive controls O-1966 (VCG-0120) and O-2052 (VCG-01213). From the 187 isolates, 130 were pathogenic to banana and 57 were endophytic *F*. *oxysporum* (S1 Table). The primer set *Foc*TR4-F/*Foc*TR4-R, targeting *Foc* TR4, also resulted in positive amplifications when DNA from the five *F*. *oxysporum* endophytes EC5-LR-GM1, CAV017-EO, EE-E-03, EE-E-06, CAV052-EO was used (Fig 1B). No amplification was observed with the primer set W2987-F/W2987-R [33] that targets *Foc* TR4, except for the positive *Foc* TR4 control.

### Genetic relationship among *F*. *oxysporum* isolates from Ecuador and *Foc* lineages

**Fig 2:**
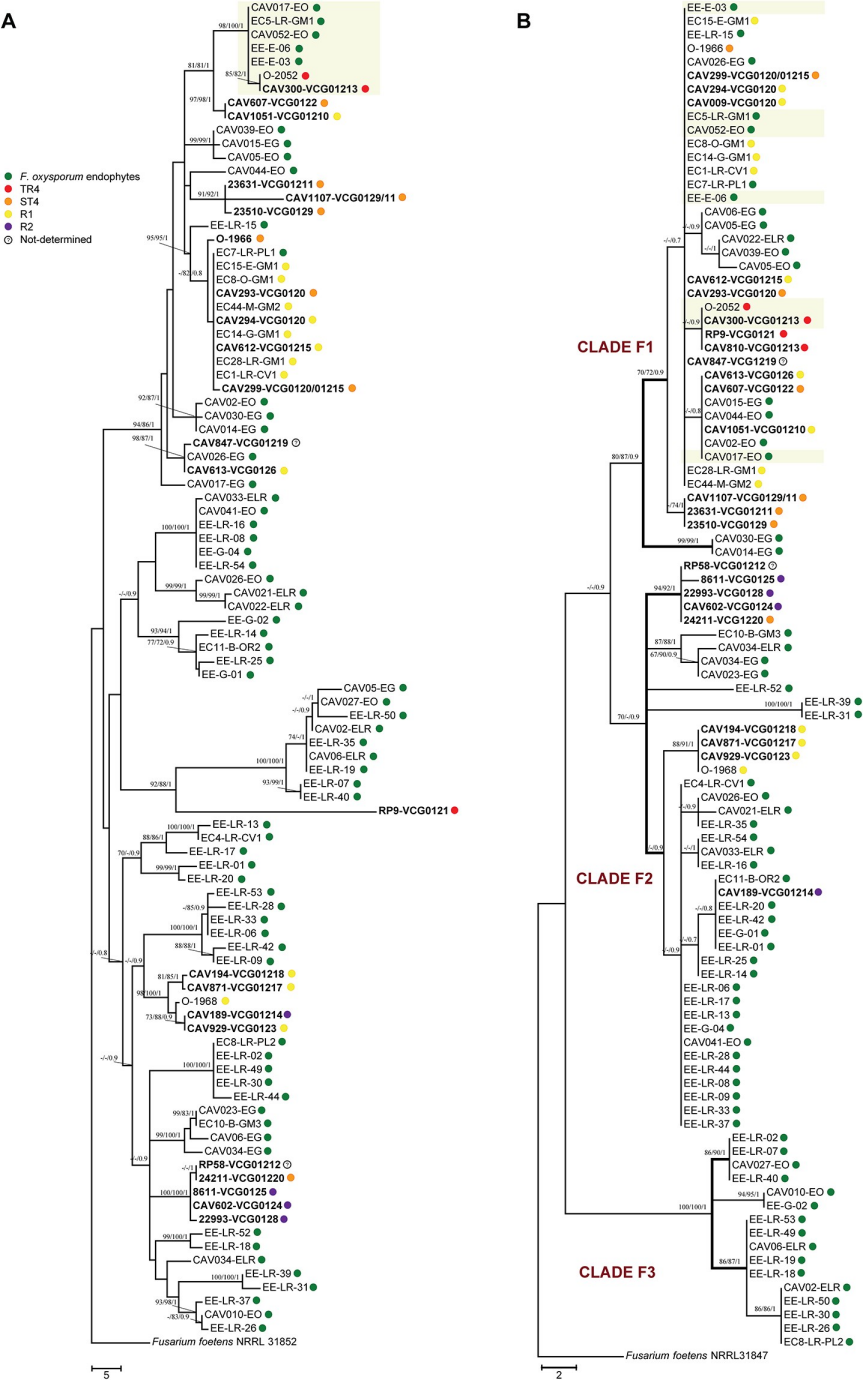
Phylogenetic relationships among *Fusarium oxysporum* isolates associated to banana. (A) Most parsimonious tree (CI: 0.67; RI: 0.93) inferred from the IGS region showing the interrelationship of pathogenic and endophytic strains of *F*. *oxysporum*. Races of *Foc* and endophytic isolates are colored designated. The shadowed area emphasizes the isolates that generated false positive amplifications, grouped to the reference *Foc* TR4 strains (red dots). (B) One of nine most parsimonious trees (CI: 0.90; RI: 0.99) inferred from the TEF region placing *F*. *oxysporum* isolates from banana in three phylogenetic clades. Previously described pathogenic lineages of *Foc* [19] are in bold. Endophytic isolates associated with false positives (marked in green) differed with those of *Foc* TR4 represented in a single lineage (marked in red) according to TEF. Bootstrap values and posterior probabilities are shown in each tree.

Selected isolates from the collection were employed to assess their phylogenetic relationship with previously described *Foc* lineages (Fig 2). The TEF dataset consisted of 100 taxa and 605 total characters, of which 54 were parsimony informative. All analyses (MP, ML and BS) resulted in three well-supported clades named as F1, F2 and F3 (Fig 2B). Endophytic *F*. *oxysporum* were distributed across the three clades. *Foc* genotypes were present only in clades F1 and F2, including *Foc* isolates EC15-E-GM1, EC44-M-GM2, EC14-G-GM1, EC28-LR-GM1 and EC8-O-GM1 from Ecuador regarded as race 1. The endophytic isolates that generated a positive PCR amplification for *Foc* TR4, using the primers *Foc*TR4-F/*Foc*TR4-R, where closely related to each other based on IGS. The isolates EC5-LR-GM1, CAV052-EO, EE-E-03 and EE-E-06, displayed the same TEF sequence and clustered together with pathogenic strains belonging to VCGs 0120, 01215, 01219, as well as with pathogenic isolates from Ecuador (Fig 2B). The isolate CAV017-EO was placed in a sub-clade together with isolates from VCGs 0126, 0122, and 01210, and with others *F*. *oxysporum* endophytes. None of the endophytes above mentioned were closely related to *Foc* TR4 strains based on the TEF phylogeny.

The IGS sequence data set consisted of 100 taxa and 1,324 characters in total, of which 187 were parsimony-informative sites. The IGS phylogeny presented a different tree topology compared to the TEF phylogenetic tree. The five endophytes EC5-LR-GM1, CAV017-EO, EE-E-03, EE-E-06, CAV052-EO that generated positive PCR amplifications for *Foc* TR4 using primers *Foc*TR4-F/*Foc*TR4-R, were very closely related to *Foc* TR4 strains associated to VCG-01213 according to this region (Fig 2A). Sequence comparison of the entire IGS region (approximately 2,431 bp), revealed a 99.8% sequence similarity of all five endophytes with the reference *Foc* TR4 strain. Differences included five Single Nucleotide Polymorphisms (SNPs) from which one was an insertion at the position 334 (T) and four-point mutations at positions 702 (A/C), 820 (T/C), 1405 (T/G) and 1560 (G/A).

### VCG analysis and pathogenicity tests

*Nit*-mutants from the five endophytes, EC5-LR-GM1, CAV017-EO, EE-E-03, EE-E-06, CAV052-EO, were not compatible with the tester strains O-2052 (VCG-01213), O-1966 (VCG-0120) and O-1968 (VCG-0123), belonging to *Foc* TR4, STR4 and race 1, respectively (Fig 1C). Self-compatibility of the tester strains was corroborated as a control measure by pairing *nit*-1 and *nit*-3 mutants with nit-M mutants of each strain resulting in a dense aerial growth at the contact zone in minimal medium. Fusarium wilt symptoms were not observed in any of the banana plants, either ‘Gran Naine’ or ‘Gros Michel’, inoculated with each of the five endophytes, EC5-LR-GM1, CAV017-EO, EE-E-03, EE-E-06, CAV052-EO, even at 30 days post-inoculation (Fig 1E and Table 2). Cross sections of pseudostems and rhizomes of all plants inoculated with spores of the five referred isolates did not show necrotic lesions, however *F*. *oxysporum* colonies were recovered from root and rhizome samples from those plants when plated on Nash-semi selective media at the end of each experiment. Plants used as positive controls started to show yellowing and wilting symptoms on the lower leaves from day 15, which later progress to all leaves. By the end of the experiment, the lower leaves of these plants were completely necrotic, and some detached from the pseudostem.

**Table 2:** Pathogenicity tests and PCR screening with total genomic DNA from inoculated banana plants.

Treatment		Pathogenicity test		PCR screening with total genomic DNA from inoculated banana ^a^					
Cultivar	Isolate	Severity^b^	DI^c^	Tissue^d^	Nested PCR-IGS/*Foc*TR4	*Foc*TR4-F/ *Foc*TR4-R	*Foc*-F/*Foc*-R	*Foc*1/*Foc*2	W2987F/ W2987R
'Gros Michel'	EC5-LR-GM1	0.0 ± 0.0	0	R	6	3	1	1	0
				P	6	0	0	0	0
	CAV017-EO	0.0 ± 0.0	0	R	6	0	2	0	0
				P	4	0	1	0	0
	CAV052-EO	0.0 ± 0.0	0	R	6	2	3	1	0
				P	5	1	0	0	0
	EE-E-03	0.0 ± 0.0	0	R	6	2	3	1	0
				P	5	0	1	0	0
	EE-E-06	0.0 ± 0.0	0	R	6	0	3	0	0
				P	5	0	0	0	0
	O-2052 (TR4) Positive control	3.83 ± 0.2	76,6	R	6	6	4	5	6
				P	6	3	4	5	6
	O-1968 (R1) Positive control	2.67 ± 0.3	53,3	R	0	0	0	0	0
				P	0	0	0	0	0
	Negative control (water)	0.0 ± 0.0	0	R	0	0	0	0	0
				P	0	0	0	0	0
Cavendish 'Gran Naine'	EC5-LR-GM1	0.0 ± 0.0	0	R	5	4	2	0	0
				P	7	3	0	0	0
	CAV017-EO	0.0 ± 0.0	0	R	7	2	2	0	0
				P	8	0	6	0	0
	CAV052-EO	0.0 ± 0.0	0	R	5	2	0	0	0
				P	0	0	0	0	0
	EE-E-03	0.0 ± 0.0	0	R	8	0	0	0	0
				P	2	0	0	0	0
	EE-E-06	0.0 ± 0.0	0	R	7	1	1	0	0
				P	4	1	0	0	0
	O-2052 (TR4) Positive control	4.25 ± 0.3	85	R	8	8	5	4	8
				P	8	8	2	3	8
	O-1968 (R1) Negative control	0.0 ± 0.0	0	R	0	0	0	0	0
				P	0	0	0	0	0
	Negative control (water)	0.0 ± 0.0	0	R	0	0	0	0	0
				P	0	0	0	0	0

^a^ Values represent the number of samples with positive PCR amplification for race 4 out of the total number of samples evaluated from two experimental replicates. In total, six plants per treatment were tested for ‘Gros Michel’ and eight plants for Cavendish ‘Gran Naine’.

^b^ Disease severity based on the discoloration of the rhizome using a scoring scale; where 0 = no discoloration, 1 = 1–15% discoloration, 2 = 16–33% discoloration, 3 = 34–50% discoloration, 4 = more than 50% discoloration and 5 = totally decayed. (NS) = no symptoms.

^c^ Disease Index (DI) according to McKinney’s formula where DI = [Ʃ (score in the scale x frequency)/(total number of plants x maximum class in the scale)] x 100. Values derived from two replicates.

^d^ R, rhizome; P, pseudostem.

### Tests on plant and soil material

Similarly to the results obtained using pure fungal DNA, positive PCR amplifications reactions were also obtained from the rhizome and pseudostem of banana plants inoculated with EC5-LR-GM1, CAV017-EO, EE-E-03, EE-E-06, and CAV052-EO isolates. However, higher number of positive PCR reactions were generated with the nested-PCR approach implemented with the primer set *Foc*TR4-F/*Foc*TR4-R (Table 2). Reactions carried out with the primer set W2987-F/W2987-R did not yield any band amplification, except for the positive controls (Table 2). The screening of soil samples derived from pathogenicity tests, using the same four primers sets as described earlier confirmed results obtained from DNA from mycelium, and from inoculated plant material. Positive PCR amplifications were also obtained for soils inoculated with endophytic isolates EC5-LR-GM1, CAV017-EO, EE-E-03, EE-E-06, CAV052-EO, for primes sets FocTR4-F/FocTR4-R, Foc-F/Foc-R, and Foc1/Foc2, but not for W2987-F/W2987-R, which only generated positive amplifications with soil inoculated with isolate O-2052 (TR4). As expected, soil inoculated with isolate O-2052 (TR4) generated positive amplifications with all primer sets, whereas soil inoculated with isolate O-1968 (R1) generated positive amplifications only with TEF primers, used as control. No positive amplifications were detected in non-inoculated soil.

## Discussion

The recent worldwide outbreaks of Fusarium wilt caused by *Foc* TR4 have proven to be a trans-continental threat of special importance for countries from central and west Africa, the Indian subcontinent and the Americas, where bananas are considered a valuable source of nutrients and calories for the subsistence of families and an important commodity crop [15,59]. For Latin America countries, a contingency plan for an eventual *Foc* TR4 outbreak was recently developed by the Regional Organization for Plant and Animal Health (OIRSA) in an effort to provide guidelines to National Plant Protection Organizations (NPPOs) on how to deal with a putative incursion of *Foc* TR4 in the region, including diagnostics, eradication and contention measures. Given the high risk that *Foc* TR4 represents, early detection and accurate diagnosis play a key role for the implementation of these strict quarantine measures upon detection of new outbreaks of the pathogen. In this regard, and equally important, a false positive is as potentially alarming and devastating for the banana industry as a false negative.

Over the last decades, all efforts to develop molecular markers for detection of *Foc* race 4 strains, including STR4 and TR4, have predominantly focused on the use of pathogenic strains [30–38], which in our view are just a small fraction of the population diversity; outliers rather than representatives of the species [44–46]. The high diversity present within the *F*. *oxysporum* species complex (FOSC), which has been underestimated [46], may pose a risk for such markers compromising their specificity; even more if within such diversity may be nonpathogenic strains, which could also contain the same genomic regions exploited for detection. Here, we describe the cross-reactivity of pathogenic and nonpathogenic *F*. *oxysporum* isolates from different banana cultivars from Ecuador with specific detection markers that target race 4 strains of *Foc* and highlight how diversity matters to avoid false positives. We also emphasize and encourage the adoption of a more holistic, ecological-based view of *F*. *oxysporum* during the process of developing PCR molecular-based detection methods by taking population diversity, including pathogens and endophytes, into account.

Comparisons between experiments confirmed that three out of four PCR methods are not reliable in detecting the taxa for which the methods were developed. Two of them, developed to detect *Foc* race 4 strains, including STR4 and TR4, cross-reacted with pathogenic isolates from Ecuador, as well as, with nonpathogenic isolates. A plausible explanation, for the cross reaction with pathogenic isolates of our collection, is that in *Foc* different pathogenic phenotypes are associated with strains of the same or similar genotypes found within the same VCG group [31]. An excellent example comes from the strains associated with VCG-0120. Strains of this VCG that cause disease on Cavendish in the subtropics may be classified as STR4, but in the tropics, where they are unable to cause disease on Cavendish, such as the case of Ecuador, are classified as race 1. This issue was observed in previous studies [31] but has now been further validated with our results. This raises concerns about the specificity of other methods proposed for the detection of race 4 strains [32, 34, 36, 38] developed based on the same RAPD sequence fragment OPA02_404_ used by Lin et al. [30]. This marker was present in all isolates analyzed in this research. It is possible that the genomic regions used for the design of these primers, although thought to be exclusive to certain pathogenic strains, are part of the ‘core genomic’ regions also found in nonpathogenic isolates. These ‘core genomic’ regions contain genes essential for the growth and survival of the fungus, and unlikely to be associated with host-specific pathogenicity [60]. Therefore, they should not be relied upon to accurately identify pathogenic variants. Putative pathogenicity genes are present in ‘accessory genomic’ regions, which can be horizontally transferred in small supernumerary chromosomes [61]. Another explanation for these faulty diagnostic markers is that the diversity of *F*. *oxysporum* has been underestimated. Populations of *F*. *oxysporum* from soil and in association with plants as endophytes have been previously reported to be highly diverse [44–46]. However, the diversity of isolates used for the development and validation of these methods did not include endophytes from banana.

The primer set *Foc*TR4-F/*Foc*TR4-R, commonly used for quarantine procedures and *Foc* TR4 diagnostics [23], also cross-reacted with five *F*. *oxysporum* endophytes from Ecuador. These endophytes presented highly similar sequences of the IGS region with *Foc* TR4 strains. However, further phylogenetic comparison using TEF, a housekeeping gene commonly used to reflect the evolutionary history of *Foc* [19, 17, 62], showed that the same isolates were not related to *Foc* TR4 strains. For these reasons, we recommend that the regions exploited for the above-mentioned diagnostic markers, including the IGS, should no longer be used for detection of race 4 strains. Conversely, the primers developed by Li et al [33] accurately discriminated *Foc* TR4 from our complete collection, including pathogenic and endophytic isolates. This set of primers has also been shown to discriminate other pathogenic strains associated with other races of *Foc* [33]. This test represents a more reliable alternative for *Foc* TR4 strain identification since it was developed on a region putatively related to virulence [33]. Moreover, this same region has been further exploited for the detection of TR4-like strains, not only associated to VCG-01213 but also to VCG-0121 and proved to discriminate nonpathogenic isolates from a different country as well [63].

Our study assessed the results from the molecular screening more in-depth by performing additional testing methods, including VCG and pathogenicity tests. Although the use of these methods has been highly criticized due to their laborious and time-consuming nature [20, 31], their inclusion allowed us to confirm that the five endophytes that displayed positive results using the *Foc*TR4-F/ *Foc*TR4-R primer set, were not true *Foc* TR4. Based on our current findings, continuous *in vitro* testing for pathogenicity and VCG analysis should not be completely disregarded. Confirmatory tests should not be the exception but rather the norm, and thus used in parallel to determine a method’s robustness.

Our results also showed that false positives can occur if infected plant tissues and soil were the matrix used for detection. The low frequency of positive PCR reactions obtained from infected plant tissue, from the total number of samples tested in our work, may have been due to the low amount of fungal DNA present in the asymptomatic tissue taken for analysis. This hypothesis was confirmed with a higher frequency of positive PCR reactions obtained with a more sensitive approach (Nested-PCR). It is known that *F*. *oxysporum* is a good colonizer of roots and stems of plants [43, 46]. After initial infection of the root cortex, the passage of *F*. *oxysporum* to xylem vessels of plants and systemic growth may depend on the levels of quantitative resistance present in the host [29]. It is not clear if the colonization events detected by PCR in the pseudostem of plants occurred through colonization of the vascular tissue (xylem vessels) or were restricted to the parenchyma tissue or epidermis. Likewise, it is unknown if such colonization was due to host-specific interactions, or other predisposing conditions that might have hampered the defense system of the host and facilitated the colonization to upper parts of the plants. Despite this uncertainty, the largely unexplored, high diversity of *F*. *oxysporum* present in the soil and the roots of bananas plants [64], makes us wonder if similar genotypes to the ones found in this study could be also found in other geographic areas that may lead to false diagnosis. We speculate that this hypothesis might be feasible based on the recent occurrence of false positives obtained in one banana farm from Australia on March 28, 2015 [65]. The Mareeba farm was closed on April 3, 2015, after a positive *Foc* TR4 PCR reaction was obtained from the tissue of a Cavendish banana plant displaying wilting symptoms. The farm remained quarantined despite being found later that *Foc* TR4 was not present in the farm [65, 66]. We hypothesize that endophytes of *F*. *oxysporum* were present in the sample and cross-reacted with the primers tested. The Mareeba farm, as well as another banana farm in Tully, also impacted by the quarantine response to *Foc* TR4, have now been reimbursed a combined total of US $684,243, which was the net value forgone for the period in which they were unable to trade [67].

## Concluding remarks

The recent outbreaks of *Foc* TR4 in new geographic locations suggest that pathogen dissemination will likely to continue if quarantine measures are inadequate [22, 23]. Thus, accurate identification of the pathogen is likewise necessary for activities related to successful quarantine, eradication, contention, and communication with banana producers at regional, national and international level. As shown in our work, one roadblock to the management of this pathogen is the generation of false positives that occur during the identification process. This is due not only to poor handling of the samples but also to the presence of non-targeted strains of *F*. *oxysporum* that are genetically similar but lack true pathogenicity. Yet, standardized protocols should be put in place to minimize the risk of contamination and favor reproducibility [63, 68]. This should be a warning for international regulatory agencies involved with plant sanitation and crop-protection. Surveillance procedures in soil and asymptomatic tissues, in areas where the pathogen is not yet reported that rely on currently accepted PCR-based detection methods for *Foc* TR4, should be carried out with caution or avoided. A better understanding of the population structure of *F*. *oxysporum* in each location should be a requirement before the adoption of any technology to minimize the risk of false positives. In the meantime, we recommend the use of the primer set W2987 F/ W2987 R [33] for identification of the VCG-01213, or the newly optimized FWB-TR4 test for detection of VCGs 01213 and 0121 [63], as molecular tools in the process of *Foc* TR4 diagnosis. Still, it is important to considerer that no technology is absolute, and no single method is completely reliable, as they all are impacted by the biology of the pathogen and its interaction with the host and environment [68]. For future diagnosis of *Foc* TR4 and to inform decisions, we recommend that each event should be analyzed in a systematic manner to enable an accurate interpretation of results, and to take a conservative approach.

Finally, race designation in *Foc* should be also reconsidered. Given the evidence of the acquisition of transferable elements between individuals within the species and the high genetic diversity characteristic of this group, the best tactic for characterizing pathogenic phenotypes should be based on strain designation rather than race approach [69]. This view is concurrent with the recent appraisal of pathogen regulation [70], where a gene-based biosecurity system proposed could more accurately assess biological risk than the conventional name-based current practices [70].

## Supporting information

S1 Table Isolates of *Fusarium oxysporum* and other *Fusarium* spp. used in this study. List of isolates used in this study, sampling information, and PCR amplification results using PCR-based identification methods for *Foc* race 4.
(XLSX)
